# Please don't gayify!: an autoethnographic account of medicalised relationality for LGBTQI+ safe affirming medical health education and clinical practice

**DOI:** 10.3389/fsoc.2024.1438906

**Published:** 2024-08-21

**Authors:** Mark Vicars, Mickey Deppeler

**Affiliations:** Department of Education, Victoria University, Melbourne, VIC, Australia

**Keywords:** LGBTQ+, health care, heteropatriarchy, misgendering, autoethnography, medical model

## Abstract

In this article, the authors, a cis-gender gay man and an Indigenous non-binary, two-spirit person, narrate their past encounters with health professionals and their experiences pursuing allied health care training as students. Taking an autoethnographic approach, the first author re-narrates how medical practitioners and students engage (or fail to engage) with the LGBTQIA+ community. They draw on gray documentation derived from an interaction with a consulting physician that highlighted a telling lack of knowledge about the LGBTQ+ community, including those with diverse sex characteristics and sexualities/manifesting as unconscious bias. This interaction provided the impetus to speak back to the experience of being reduced to a medical prognosis. The second author questions the hegemonic practices underpinning encounters with the medical model of response in tertiary education. Our remit in this paper is to question how adequately the specific needs of the LGBTQI+ population are being addressed by the medical model and to what extent aspiring clinicians understand how their actions can contribute to gender- and sexuality-based discrimination and stigmatization.

## Introduction: leave your body at the door

In this article, we draw from our lived experience to articulate how the intersectionalities within LGBTQI+ communities are often neglected in allied health professionals' education and clinical practice. Valentine (2007) has noted that the constantly changing complexities of negotiating intersectionality require an awareness of how the LGBTQI+ population is constituted across a wide spectrum of cultural identities, genders, religions, ethnicities, impairments, ages, and disabilities. This article, with a particular focus on the Australian context, examines prejudice in medical healthcare education and clinical practice. As we reflect on the material and situational structures of our lived experiences, which visibly express our queer positionalities, we address the issues within healthcare domains. Too often, encounters and interactions with the medical profession make queer individuals feel like unwanted guests at a dinner party.

Fear of prejudice is already known to impact the numbers of LGBTQI+ community members seeking healthcare provision (Asztalos et al., 2009; Olson et al., 2016; Abreu et al., 2022).

It has been claimed that health outcomes for LGBTQI+ individuals are poorer in comparison to their heterosexual counterparts (Poteat et al., 2021). In the state of Victoria, Australia, alarming statistics have been published that indicate that the healthcare system is failing LGBTQI+ community members in all areas (Gil et al., 2021).

A study by Stewart and O'Reilly (2017) showed that in aged care, transgender and non-binary adults were victims of continuous misgendering, inappropriate comments, abuse, and deadnaming. Copeland et al., 2023, in “Creating Change with Families: Reflections and Recommendations for the Care of Gender Diverse and LGBTQIA+ Individuals and Their Families Throughout Pregnancy and Birth,” referenced two separate members of the queer community (one who identified as trans and the other as non-binary) who expressed their fear of misgendering. Russ (2018) noted the harmful effects of “under the breath” comments from midwives when assisting LGBTQI+ individuals. Considering the increase in what Nash and Browne (2020) coined as heteroactivism, a reinvigorated global opposition to LGBTQ+ equalities and rights, this article aims to reconsider how LGBTQI+ inclusion can be better integrated into the allied health landscape and clinical practice.

The shame of being/becoming the other within the quotidian discourses of medicalized relationality provoked us the following wonder. How is it that the LGBTQ+ body continues to be habitually inscribed by epistemic practices that fail to acknowledge the experiential intersections of ethnicity, cisgender, class, sexual identity, social background, age, and visible and non-visible impairments? As we started to question how adequately the tertiary healthcare curriculum addresses the specific needs of the LGBTQI+ population, our focus shifted to how aspiring clinicians understand the routine experiences of discrimination and stigmatization encountered by LGBTQI+ individuals. What barriers prevent the provision of safe and affirming care to LGBTQI+ clients? Does it always have to be us that has to be deconstructed?

To think differently about these questions is to think with agency and resist the tragic trope. It is to affirm that experiences arising from embodiment are critical to a range of civil, political, economic, social, and cultural rights. Too often, we have to engage in self-translation that requires a confessional narrative of trans, gay, and genderqueer personhood; we suggest this should not only be understood as a psychological phenomenon but also as a sociological occurrence. We call for rescripting healthcare education and practices that move beyond dualist thinking. This consultative approach should involve a somatic reorientation of fluidity, liminality, and abjection as a performative signifier of existence.

This often requires health practitioners to possess an understanding of the ever-evolving ecology of multifaceted coded meanings that exist within LGBTQI+ communities, which requires constant review, reconsideration, and re/textualization.

## Not another autoethnography? Do you really want to know me? Do you really care?

We acknowledge that our bodies and hearts carry trauma and weariness from daily struggles for survival and self-care, but they also carry defiant dreams. For us, autoethnography must necessarily be a generative and enabling frame for exploring the possibilities borne of our defiant dreams (Bell et al., 2020, p. 851).

When a narrative is entrenched, disruption and provocation are required. Reflecting on our lived experiences, we (re)construct our quotidian experience of the doxa of dis/connections and signpost for the reader that in our reconstructed accounts, we do not separate what we know from how we know (Ahmed, 2006; Seigworth and Gregg, 2010).

Our autoethnographic approach examines complex medical infrastructure, and Kincheloe (2004) has likened autoethnography to a form of critical pedagogy in its commitment to transformative and emancipatory processes. In its pursuit of situated understandings, autoethnographic accounts can elucidate how self-construction emerges from a set of relationships.

Thus, in an ontological context, meaning emerges not from the thing itself but from its relationships to an infinite number of things. Furthermore, autoethnography has the potential to capture the passion, feelings, and struggles of personal experiences on the page (Ellis and Bochner, 2000).

Researchers bring various experiences, scholarship, and theoretical and practical understandings to research. Epistemological considerations are not abstractions, and autoethnographic accounts that have addressed diverse genders and sexualities have framed how being visibly LGBTQ+ in medical domains can mean becoming subject to the ideological and performative functionality of the default assumption of heteronormativity (Speciale et al., 2015; Popova, 2017; Ozalas, 2020; Suárez, 2022; Johnson et al., 2024).

## Storying as autopoiesis: a counter-hegemonic tactic

In the end, we all become stories—Margaret Atwood.

Human action is inherently interpretive, as all actions carry meaning and intention, existing within a social and cultural context that further attaches meanings to them. Any attempts to interpret these actions and events must reference their already-interpreted nature, as well as the background of intention and conventional understandings, which must also be reinterpreted.

Critically reflecting on his experience(s), Author 1, who identifies as a cis-gender gay male, recounts an accident of being admitted to an emergency with a suspected case of viral meningitis. He acknowledges that he does not conform to normative masculinity and is acutely aware of how he is perceived by the disciplinary imagination of the straight social world. Through an epistemology of proximity, he re-stories a narrative of psychological, physical, and psychic dis/ese, illustrating how being is always subject to ideological and performative functions and how heteronormativity is privileged by default.

*I remember little of my ambulance admission to A&E for suspected meningitis but I do remember the aging White, male, assumed heterosexual* ***treating physician insisting I*** ***complete a questionnaire that inquired of activities associated with non-heterosexual*** ***sexual orientations**. I have cut and pasted sections from a letter that was written by the physician on my release from hospital and unknowingly to me was sent to my Dean for processing on to Human Resources as I was due to fly overseas for work in the following 3 days and my University requested assurance for insurance purposes that I was well enough to travel*.

*22 May 2014*

*Dear Richard*

*Re: Dr Mark Vicars*

*Dr Mark Vicars was admitted to Geelong hospital under my care on Wednesday, 21 May. Last Thursday when he returned home from work at about 9 PM, he felt hot and sweaty, approximately 5 min later he developed the sudden onset of a severe headache over the vertex and down the side of the head bilaterally but not frontal or occipital. The headache was 10/1 0 severity. He felt febrile over the next few days, and on a Saturday he developed nausea and vomiting, he was photophobic, he didn't experience phonophobia because there was no noise where he lives. There were no visual or neurological symptoms. His friend forced him to come to the hospital when she rang an ambulance on the Wednesday, he simply lay in bed for the 4 days, there was some neck stiffness. He has a past history of insulin-dependent diabetes approximately 9 years, glaucoma and coronary artery disease. His full blood examination and CRP were both normal. He is a lecturer at Victoria University, he smokes 20–25 cigarettes per day, drinks alcohol, does not use intravenous drugs but is at risk of HIV because of his homosexuality in the setting of not*.

*I think he most likely had a viral meningitis, the rigor preceding the headache and the focal nature the headache would mitigate against the diagnosis of a subarachnoid hemorrhage. Sometimes with viral meningitis one can see the sudden onset of a severe headache. I did suggest however that at some stage he probably should have a HIV test although I think this is unlikely to have been HIV infection, it is recommended that HIV tests should be done on a regular basis. The purpose of this letter is for you to keep it and use it in future consultations with other Doctors. This letter represents what I understood from your recent consultation. Could I suggest that it would be a good idea if the patient carried this letter with him when he travels as this would greatly assist any Doctors he might would need to see*.

*Regards*

*Professor bmbmbmb*

I am forever grateful that the letter was intercepted and “lost” by my Dean at the time, preventing it from reaching Human Resources and being added to my personal employment file. Since 2014, I have kept the letter, unsure of what to do with it. Now, I interpret it as an example of institutional sacred textualities that shape and inform performative narratives around identity, power, and social being. I have come to understand that clinical practice often privileges dominant ideologies, embedding them as routine regulatory practices of subjectification. Each time I read and re-read that letter, it highlights the absence of LGBTQ+ sensitivity exhibited by the attending physician during my hospital admission.

## Praxis makes perfect: LGBTQI+ inclusive practice in healthcare settings

Reflecting on the experiences of the second author, an Indigenous non-binary, two-spirit, person, and a student in Allied health education, we see a vivid illustration of the paralysis encountered when formal curriculum content dedicated to LGBTQI+ people is absent. The second author aimed to educate fellow students that, at some point in their careers, they would likely encounter individuals who are transitioning and need to know how to communicate effectively with pre-op or post-op patients. This involves understanding the psychological assessment required for trans, non-binary, and gender-fluid communities.

When the second author decided to incorporate more LGBTQI+ inclusive content into group presentations and case studies, altering statistics and medication regimens to reflect individuals in various stages of transitioning, they faced significant pushback. Classmates frequently asked, “Why are you making the content gay?” or, in one instance, “Please don't gayify the presentation,” in a course that offered few formal learning opportunities to prepare students to work with LGBTQI+ clients.

Critically reflecting on Zir's experience as a healthcare student, they highlighted the frequent misconceptions and contradictions related to LGBTQI+ individuals among staff and students. They suggested that the tools for understanding are lacking in clinical settings due to the absence of relevant education or support (Block, 2014). This lack of education, they argued, begins in tertiary educational settings and can easily flow into clinical practice, where bi-gender markers remain the default.

The second author notes that without essential education on how to interact with minority groups and using correct nomenclature when assessing or interacting with gender-diverse or LGBT patients, LGBTQ+ individuals will likely remain in a state of mental exhaustion. They argued that allied health education at a tertiary level is not gender-queer inclusive. Only when specialists work in facilities with LGBTQ+ inclusive practices will they receive the necessary training to assess clients properly.

At a tertiary level, the allied healthcare curriculum can be actively criticized for its lack of LGBTQ+ inclusivity and diversity. For example, most medical textbooks feature pictures of white cis men and women, perpetuating systemic binary gender norms (Kirjava et al., 2023).

## To be or not to be out: that is the question

Chapman et al.'s (2012) study found that parents in a homosexual relationship often choose not to disclose their orientation to health professionals due to fear that their children might not receive proper care. Table et al. (2022) highlighted a lack of compassion for individuals identifying outside binary norms. Additionally, Roth et al. (2020) developed a course for 4^th^-year medical students examining their readiness for residency. Many students reported a clear lack of training dedicated to LGBTQI+ healthcare; when training was available, it was often incorporated into existing modules without significant professional impact.

To ensure that stories like ours are not continuously repeated, it is essential for more LGBTQ+ individuals to report their encounters. Data can foster change and enable the recovery of “excluded subjects” within and through discourses that promote the values of normativity (Tamboukou and Ball, 2003, p. 5).

Data serve as an ontological-ethical-epistemic space from which to reconsider forms of public knowledge-making and argumentation (Vicars and Arantes, 2023). In the 1980′s, it was not until the Centre for Disease Control in America began recording data on “innocent victims” that the political implications of HIV+ were acknowledged, leading to greater scrutiny of how new cases were counted. The resulting data categorized belonging, which subsequently funded medical research and designated HIV+ as an LGBTQ+ issue, with the LGBTQ+ community orchestrating responses to the increasing rates of infection.

The free-floating signifier of the rainbow flag, designed by Gilbert Baker in 1978, became a symbol of pride and defiance. This was further augmented in 1987 with the creation of the NAMES Project: the AIDS Memorial Quilt, which announced to the world: “we're here, we're queer, and we're dying.” The quilt, consisting of 48,000 panels, covers three acres of land when unfolded. It visually represents the effects of HIV and has become a powerful queer political symbol and form of data that continues to be sustained by the community.

At the most basic level, allowing the smallest signifier, such as a rainbow flag or an ally sign, gives someone entering a clinical setting a sense of security. It signals that the space is a safe environment for the LGBTQ+ community and that it is okay to reveal a part of their identity. The second author notes how these signs can create an atmosphere of acceptance and understanding. These are his remarks:

*During one of my placement I found myself drawn to the physiotherapist who wore pronoun pins and ally socks. Seeing these signifiers gave me the permission to be more myself. I was able to engage more and more because I didn't have a continuous anxiety of anticipating homophobic comments*.

## Disappointments and dissonance…That can't be the only story!

The RACGP^1^ reports that 65.5% of those who identify as LGBTQI+ do not have a regular GP due to fears of not receiving proper care. Muntinga et al. (2015) conducted a study evaluating biomedical and sociocultural aspects of diversity and found that learning objectives in medical knowledge, skill, patient–practitioner communication, and reflexivity were somewhat lacking.

Wilby et al. (2022) noted the need to dismantle systemic discrimination in areas such as pharmacy education and practice. In critiquing the medical discourses in our lived experience, the second author observed that when students would complain or question why they were “gayifying” the presentation, the real “problem” was how the allied health curriculum was informed by and connected to wider social, cultural, and historical heteronorming forces.

The psychosocial risk of healthcare policy and practice for LGBTQ+ communities in clinical healthcare can become riddled with affective relations connected to intrinsic views of selfhood, self-esteem, efficacy, and agency (Foucault, 1994).

## Limitations

We suggest there is a cultural need for greater gender and sexual orientation training for allied health professionals and in clinical practice. However, how can this be accomplished when it is not a priority for educational institutions or accreditation organizations? By synergizing an *a posteriori* knowledge of heteronormative practices in tertiary educational and clinical domains, we can address how specific phenomena are explored in temporal and spatial settings that we claim remain under investigated. Our encounters with the medical model and discourse have taught us how the body, for LGBTQ+ identifying individuals, can be a site of betrayal. In our reconstructed account, we hope the verisimilitude and legitimacy of our experience may connect with first-hand familiarity.

The vignette in this article required us to “transform lived experience into a textual expression that is not a reflective re-living and a reflective re-appropriation of something meaningful” (van Manen, 2016, p. 36). While Adams (2015) recognize that stories shift with “time, space, and context” (95), Sikes and Goodson (2017) declare that “it is through the [re]construction, telling, and retelling of personal stories, to ourselves and to others, that we attempt to make sense of our lives and give them meaning” (61). Some memories come easily: words, sentences, and paragraphs render them sculpturesque. Others come fragmented and incomplete, necessitating time and patience for the sediments of silence to conglomerate together in recollection.

In what is not so much a conclusion but a call for an interruption in binary teleological embodied scripting, we invite a transformation of healthcare education and practices that move beyond dualist thinking. We advocate for healthcare approaches that reorient away from gender and sexual fluidity, liminality, and abjection as performative signifiers of existence. By introducing mandatory education that is relevant to LGBTQI+ lives, we may stop searching for signs in hospitals, clinics, and healthcare education that indicate it is safe to be ourselves.

## Data availability statement

The original contributions presented in the study are included in the article/supplementary material, further inquiries can be directed to the corresponding author.

## Ethics statement

Approval to conduct this research was provided by the Victoria University Human Research Ethics Committee (VUHREC).

## Author contributions

MV: Conceptualization, Data curation, Formal analysis, Funding acquisition, Investigation, Methodology, Project administration, Resources, Software, Supervision, Validation, Visualization, Writing – original draft, Writing – review & editing. MD: Conceptualization, Data curation, Investigation, Methodology, Resources, Validation, Visualization, Writing – original draft, Writing – review & editing.

## Footnotes

^1^RACG. (2021). Available at: https://www1.racgp.org.au/newsgp/clinical/lgbtqia-youth-more-likely-to-seek-mental-support.

## References

[B1] Abreu R. Townsend D. Mitchell Y. Ward J. Audette L. Gonzalez K. (2022). LGBTQ qualitative and mixed methods research in Counselling Psychology: a content analysis. Counsel. Psychol. 50, 708–737. 10.1177/00110000221092481

[B2] Adams TE, Jones, SH, and Ellis, C. (2015). Autoethnography: Understanding Qualitative Research. New York: Oxford University Press.

[B3] Ahmed S. (2006). Queer Phenomenology: Orientations, Objects, Others. Durham, NC: Duke University Press. 10.1515/9780822388074

[B4] Asztalos M. De Bourdeaudhuij I. Cardon G. (2009). The relationship between physical activity and mental health varies across activity intensity levels and dimensions ofmental health among women and men. Public Health Nutr. 13, 1207–1214. 10.1017/S1368980009992825 20018121

[B5] Bell D. Canham H. Dutta U. Fernández J. S. (2020). Retrospective autoethnographies: a call for decolonial imaginings for the new university. Qualit. Inquiry 26, 849–859. 10.1177/1077800419857743

[B6] Block B. A. (2014). Supporting LGBTQ students in physical education: changing the movement landscape. Quest 66, 14–26. 10.1080/00336297.2013.824904

[B7] Chapman R. Wardrop J. Freeman P. Watkins R. Zappia T. Shields L. (2012). A descriptive study of the experiences of lesbian, gay, and transgender parents accessing health services for their children. J. Clin. Nurs. 21, 1128–1135. 10.1111/j.1365-2702.2011.03939.x 22288982

[B8] Copeland M. Tucker J. Briley A. (2023). Creating change with families: Reflections and recommendations for the care of gender diverse and LGBTQIA+ individuals and their families throughout pregnancy and birth. Midwifery 119:103621. 10.1016/j.midw.2023.103621 36773412

[B9] Ellis C. Bochner A. (2000). “Autoethnography, personal narrative, reflexivity,” in Handbook of Qualitative Research (2nd edition), eds. N. K. Denzin, and Y. S Lincoln (Thousand Oaks CA: Sage).

[B10] Foucault M. (1994). The Order of Things. Easton: Vintage Books.

[B11] Gil R. M. Freeman T. L. Mathew T. Kullar R. Fekete T. Ovalle A.. (2021). Corrigendum to: Lesbian, gay, bisexual, transgender, and queer (LGBTQ+) communities and the coronavirus disease 2019 pandemic: a call to break the cycle of structural barriers. J. Infect. Dis. 224, 1990–1992. 10.1093/infdis/jiab408 34758068 PMC9034330

[B12] Johnson A. Mullens A. Burton L. Sanders T. Brömdal A. (2024). Pretty Bi for an Ally: a critical autoethnography. J. Bisex. 23:896. 10.1080/15299716.2024.2360896

[B13] Kincheloe J.L. (2004). “Redefining rigour and complexity in research,” in Complexity in Educational Research, eds. J. Kincheloe and K. S. Berry Rigour (Maidenhead: Open University Press).35173439

[B14] Kirjava S. A. Rawal D. Xia A. Moshin M. (2023). Contemporary LGBTQ+ content that should be included in allied health professions education. Disc. Educ. 2:6. 10.1007/s44217-023-00029-y

[B15] Muntinga M. E. krajenbrink V. E. Peerdeman S. M. Croiset G. Verdonk P. (2015). Toward diversity-responsive medical education: taking an intersectionality-based approach to a curriculum evaluation. Adv. Health Sci. Educ. 21, 541–559. 10.1007/s10459-015-9650-9 26603884 PMC4923090

[B16] Nash C.J. Browne K. (2020). Heteroactivism: Resisting Lesbian, Gay, Bisexual and Trans Rights and Equalities. London: Bloomsbury Press. 10.5040/9781350225503

[B17] Olson K. R. Durwood L. DeMeules M. McLaughlin K. A. (2016). Mental health of transgender children who are supported in their identities. Am. Acad. Pediatr. 137:3223. 10.1542/peds.2015-3223 26921285 PMC4771131

[B18] Ozalas B. (2020). “What if you meet someone else?”: an autoethnographic account of biphobia and intimate partner emotional abuse. J. Bisex. 20, 86–103. 10.1080/15299716.2020.1724229

[B19] Popova M. (2017). Inactionable/unspeakable: bisexuality in the workplace. J. Bisex. 18, 54–66. 10.1080/15299716.2017.1383334

[B20] Poteat T. Logie C. van der Merwe L. (2021). Advancing LGBTQI health research. Lancet. 397, 2031–2033. 10.1016/S0140-6736(21)01057-6 33992129

[B21] Roth L. T. Friedman S. Gordon R. Catallozzi M. (2020). Rainbows and “ready for residency”: integrating LGBTQ health into medical education. MedEdPORTAL 16:11013. 10.15766/mep_2374-8265.11013 33204837 PMC7666841

[B22] Russ S. (2018). Research shows the risk of misgendering transgender youth. ChildTrends. Available at: https://www.childtrends.org/blog/research-shows-the-risk-of-misgendering-transgender-youth (accessed November 8, 2022).

[B23] Seigworth G. Gregg M. (2010). The Affect Theory Reader. Durham: Duke University Press.

[B24] Sikes P. Goodson I. (2017). “What have you got when you've got a life story?” in The Routledge international handbook on narrative and life history, I. Goodson, A. Antikainen, P. Sikes and M. Andrews (Oxon: Routledge), 60–71.

[B25] Speciale M. Gess J. Speedlin S. (2015). You Don't look like a Lesbian: a co-autoethnography of intersectional identities in counselor education. J. LGBT Issues Couns. 9, 256–272. 10.1080/15538605.2015.1103678

[B26] Stewart K. O'Reilly P. (2017). Exploring the attitudes, knowledge and beliefs of nurses and midwives of the healthcare needs of the LGBTQ population: an integrative review. Nurse Educ. Today. 53, 67–77. 10.1016/j.nedt.2017.04.008 28448883

[B27] Suárez M. I. (2022). My Autohistoria-Teoría (trans)formational experience: an autoethnographical case study of a transgender BIPOC teacher's experience with racial healing. Int. J. Transg. Health. 23, 243–254. 10.1080/26895269.2020.1838395 35403116 PMC8986244

[B28] Table B. Tronstad L. D. Kearns K. (2022). ‘Anything is helpful': examining tensions and barriers towards a more LGBT-inclusive healthcare organization in the United States. J. Appl. Commun. Res. 50, 382–401. 10.1080/00909882.2021.1991582

[B29] Tamboukou M. Ball S.J. (2003). “Genealogy and ethnography: fruitful encounters or dangerous liaisons?” in Dangerous Encounters: Genealogy and Ethnography, eds. M. Tamboukou and S. Ball (New York: Peter Lang).

[B30] Valentine G. (2007). Theorizing and researching intersectionality: a challenge for feminist geography. Professional Geogr. 59, 10–21. 10.1111/j.1467-9272.2007.00587.x

[B31] van Manen M. (2016). Researching Lived Experience: Human Science for an Action Sensitive Pedagogy. 2 ed. Oxon: Routledge.

[B32] Vicars M. Arantes J (2023). Missing in action: queer(y)ing the educational implications of data justice in an age of automation. Learn. Media Technol. 48, 213–225. 10.1080/17439884.2023.2207141

[B33] Wilby K.j Cox D. Whelan A. M. Arya V. Framp H.. (2022). Representation of diversity within written patient cases: Exploring the presence of a “hidden curriculum”. J. Am. Coll. Clin. Pharm. 5, 837–843. 10.1002/jac5.1628

